# Comprehensively Characterizing the Cytological Features of *Saccharum spontaneum* by the Development of a Complete Set of Chromosome-Specific Oligo Probes

**DOI:** 10.3389/fpls.2018.01624

**Published:** 2018-11-06

**Authors:** Zhuang Meng, Zhiliang Zhang, Tianying Yan, Qingfang Lin, Yu Wang, Weiyuan Huang, Yongji Huang, Zhanjie Li, Qingyi Yu, Jianping Wang, Kai Wang

**Affiliations:** ^1^Key Laboratory of Genetics, Breeding and Multiple Utilization of Crops, Ministry of Education, Fujian Provincial Key Laboratory of Haixia Applied Plant Systems Biology, Center for Genomics and Biotechnology, Fujian Agriculture and Forestry University, Fuzhou, China; ^2^Texas A&M AgriLife Research, The Texas A&M University System, Dallas, TX, United States; ^3^Department of Agronomy, University of Florida, Gainesville, FL, United States; ^4^National Engineering Research Center of Sugarcane, Fujian Agriculture and Forestry University, Fuzhou, China

**Keywords:** *Saccharum spontaneum*, oligo probe, chromosome identification, fluorescence *in situ* hybridization, karyotype, chromosomal rearrangement

## Abstract

Chromosome-specific identification is a powerful technique in the study of genome structure and evolution. However, there is no reliable cytogenetic marker to unambiguously identify each of the chromosomes in sugarcane (*Saccharum* spp., Poaceae), which has a complex genome with a high level of ploidy and heterozygosity. In this study, we developed a set of oligonucleotide (oligo)-based probes through bioinformatic design and massive synthetization. These probes produced a clear and bright single signal in each of the chromosomes and their eight homologous chromosomes in the ancient species *Saccharum spontaneum* (2*n* = 8*x* = 64). Thus, they can be used as reliable markers to robustly label each of the chromosomes in *S. spontaneum*. We then obtained the karyotype data and established a nomenclature based on chromosomal sizes for the eight chromosomes of the octoploid *S. spontaneum*. In addition, we also found that the 45S and 5S rDNAs demonstrated high copy number variations among different homologous chromosomes, indicating a rapid evolution of the highly repeated sequence after polyploidization. Our fluorescence *in situ* hybridization (FISH) assay also demonstrated that these probes could be used as cross-species markers between or within the genera of *Sorghum* and *Saccharum*. By comparing FISH analyses, we discovered that several chromosome rearrangement events occurred in *S. spontaneum*, which might have contributed to the basic chromosome number reduction from 10 in sorghum to 8 in sugarcane. Consistent identification of individual chromosomes makes molecular cytogenetic study possible in sugarcane and will facilitate fine chromosomal structure and karyotype evolution of the genus *Saccharum*.

## Introduction

Sugarcane (*Saccharum* spp., Poaceae) is the leading crop in sugar production, providing 80% of the world’s sugar, and it is also an important biofuel crop in ethanol and biomass production (Wang et al., 2010). Sugarcane is currently the 5th most valuable crop worldwide, with an annual cultivation across >90 countries valued at ∼$57 billion. According to conventional taxonomy, the genus *Saccharum* typically includes six species, namely, *Saccharum spontaneum*, *S. robustum*, *S. officinarum*, *S. barberi*, *S. sinense*, and *S. edule*. Among them, *S. spontaneum* and *S. officinarum* are the two main species used in modern sugarcane breeding.

However, all of the *Saccharum* species, including *S. spontaneum* and *S. officinarum*, in this genus are complex and polyploid with highly variable chromosomal numbers. *S. spontaneum*, the most primitive species (Stevenson, 1965; Ming et al., 2010), has been reported to comprise nearly 40 genotypes, whose chromosome numbers range from 2*n* = 5*x* = 40 to 2*n* = 16*x* = 128 (Panje and Babu, 1960; Ha et al., 1999; Irvine, 1999; Grivet et al., 2006). The ‘noble’ cane *S. officinarum* typically possesses 2*n* = 8*x* = 80 chromosomes (Daniels and Roach, 1987) thus, modern sugarcane cultivars that are derived from the hybridization of these two highly polyploid species (i.e., *S. officinarum* and *S. spontaneum*) are highly polyploid interspecific hybrids. Thus, the genome complexity has hindered progress in genetic/genomic research and the application of genomic tools in sugarcane breeding programs.

Cytogenetics is a powerful tool for genome study especially in species with large sizes and complex genomes. Based on the classical cytogenetic method, researchers have established a classification of the *Saccharum* genus and, to some extent, revealed the modern cultivar “nobilization” breeding process (Sreenivasan et al., 1987; Ming et al., 2010). By a molecular technique, i.e., fluorescence *in situ* hybridization (FISH) using ribosomal DNA probes, the basic chromosome numbers in *S. officinarum* and *S. spontaneum* were determined (D’Hont et al., 1996, 1998). Moreover, FISH studies using genomic DNA as a probe revealed that 70–80% of chromosomes in current sugarcane cultivars are typically derived from *S. officinarum*, with 10–20% coming from *S. spontaneum* (D’Hont et al., 1996, 2002; Cuadrado et al., 2004; Piperidis et al., 2010). Given its relative simplicity and high cost efficiency, FISH has become an optimal approach for studies of large and complex sugarcane genomes. However, given the lack of information on genome and chromosomal morphology, no chromosome-specific FISH probes are available for unambiguously identifying individual chromosomes in sugarcane. In fact, the high levels of repetitive DNA in the genome is a huge challenge for the development of chromosome-specific FISH probes from the usual bacterial artificial chromosome (BAC) clones and PCR productions with exclusively single- or low-copy sequences (Jiang and Gill, 2006; Dong et al., 2018).

Technical advances in DNA synthesis have allowed for the massively parallel *de novo* synthesis of thousands of oligonucleotides (oligos). The major advantage of oligos is that oligos of any sequence, corresponding to any expected chromosomes or chromosomal regions, can be designed and synthesized. Thus, different types of oligo probes capable of distinguishing specific chromosomes or chromosome regions can be easily obtained (Boyle et al., 2011; Yamada et al., 2011). Recently, these massively synthesized oligos have been successfully used to label specific chromosomes or chromosomal regions by FISH in a number of plant species (Han et al., 2015; Li et al., 2016; Qu et al., 2017; Braz et al., 2018).

Here, we reported the successful designing and synthesizing oligos based on the reference genome of sorghum, which diverged from a common ancestor with sugarcane 8–9 million years ago (MYA) and retains a high level genomic synteny with sugarcane (Jannoo et al., 2007; Wang et al., 2010; Dong et al., 2018). After a FISH assay in an autopolyploid *S. spontaneum* SES208 (2*n* = 8*x* = 64), we developed eight chromosome-specific oligo probes, which can be used as cytological markers to unambiguously identify the eight *S. spontaneum* SES208 chromosomes. Karyotyping based on the chromosome identification was then conducted for *S. spontaneum* SES208. Analyses by chromosome-anchored 45S and 5S rDNAs revealed their high level of variation in copy number among homologous chromosomes. Moreover, we found that chromosome rearrangement events occurred in sugarcane, which might contribute the basic chromosome number reduction from 10 in sorghum to 8 in sugarcane. This set of chromosome-specific oligo probes allow us to dissect individual chromosomes in the complex genome crop sugarcane, which provide a valuable tool for further studies of genome structure and evolution.

## Materials and Methods

### Plant Materials and Chromosome Preparation

SES208 is an octoploid clone of the species *S. spontaneum*. The *Sorghum bicolor* inbred line BTx623 was also used in this study. All of the plants were grown in the greenhouse of Fujian Agriculture and Forestry University (Fuzhou, Fujian Province, China) with a 16-h light/8-h dark photoperiod at 30°C.

Mitotic metaphase chromosomes spreads were prepared as previously described with some modifications (Wang et al., 2007). Briefly, root tips were harvested from sugarcane and sorghum, and they were treated in 2 mM 8-hydroxyquinoline at 25°C for 2 h; then, the root tips were fixed in methanol-acetic acid (3:1) and kept at −20°C until use. An enzymatic solution with 2% cellulase (Yakult Pharmaceutical, Tokyo, Japan), and 1% pectolyase (Sigma Chemical, St. Louis, MO, United States) was used to digest the root tips at 37°C for 1 h, which were then squashed with a cover slip. After the removal of the cover slips, the slides were dehydrated with an ethanol series (70, 90, and 100%, 5 min each) prior to FISH analysis.

### Oligo Design

We used Chorus software^1^ to design chromosome-specific oligo probes as previously described (Han et al., 2015), with several modifications. We filtered out repetitive sequences in the sorghum genome by applying RepeatMasker^2^ and by dividing the remaining sequences into oligos (59 nt) with a step size of 5 nt. Then, we mapped these oligo sequences to the genome and removed oligos that map to two or more loci with 75% homology. The melting temperature Tm and hairpin Tm of each oligo were calculated, and oligos with dTm > 10 (dTm = melting temperature Tm – hairpin Tm) were kept to build an oligo pool as one probe.

### Probe Preparation and FISH

Probe preparation and FISH were conducted following published protocols (Han et al., 2015; Meng et al., 2017) with several modifications. Briefly, the oligo libraries were synthesized by MYcroarray (Ann Arbor, MI, United States). The synthesized oligos labeled with digoxin or biotin were directly used as FISH probe. The rice 5S and 45S rDNA and a sugarcane centromere-specific sequence Ss51 (Zhang et al., 2017) were labeled with either digoxigenin-dUTP (Roche Diagnostics, United States) or biotin-dUTP (Roche Diagnostics, United States) by standard nick translation reactions. For FISH, the chromosome slide was denatured in 70% formamide in 2× SSC at 70°C for 65 s. Then, the slides were dehydrated in an ethanol series (70, 90, and 100%; 5 min each). The hybridization mixture, 15 μL per slide (50 ng labeled probe, 50% formamide, 10% dextran sulfate, 1.5 μL 20× SSC) was applied to denatured chromosomes and incubated for 12 h at 37°C. Slides were washed in 2× SSC, 50% formamide in 2× SSC, and at 42°C in 2× SSC for 5 min each. Digoxigenin- and biotin-labeled probes were detected using rhodamine-conjugated anti-digoxigenin (Roche Diagnostics, United States) and fluorescein-conjugated avidin (Life Technologies, United States), respectively. Chromosomes were counterstained with 4′, 6′-diamidino-phenylindole (DAPI) in an antifade solution (Vector Laboratories, United States) under a coverslip.

Chromosomes and FISH signals were examined under an Olympus BX63 fluorescence microscope, and images were captured and merged using cellSens Dimension 1.9 software with an Olympus DP80 CCD camera. Final image adjustments were performed with Adobe Photoshop 8.0.

### Karyotype Analysis

For the karyotype analysis, 10 cells without apparent chromosomal morphological distortion were analyzed. Thus total eighty chromosome samples for each chromosome were measured. The sizes of short (*S*) and long (*L*) arms of individual chromosomes were measured, and then the arm ratio (*AR* = *L/S*), total length of each chromosome (*tl* = *S* + *L*), total length of the entire set of chromosomes (*TL* = Σ*tl*), and relative chromosome length (*RL* = *tl*/*TL* × 100) were calculated.

## Results

### Development of Oligo-Based FISH Probes for Chromosome Identification in *S. spontaneum*

**FIGURE 1:**
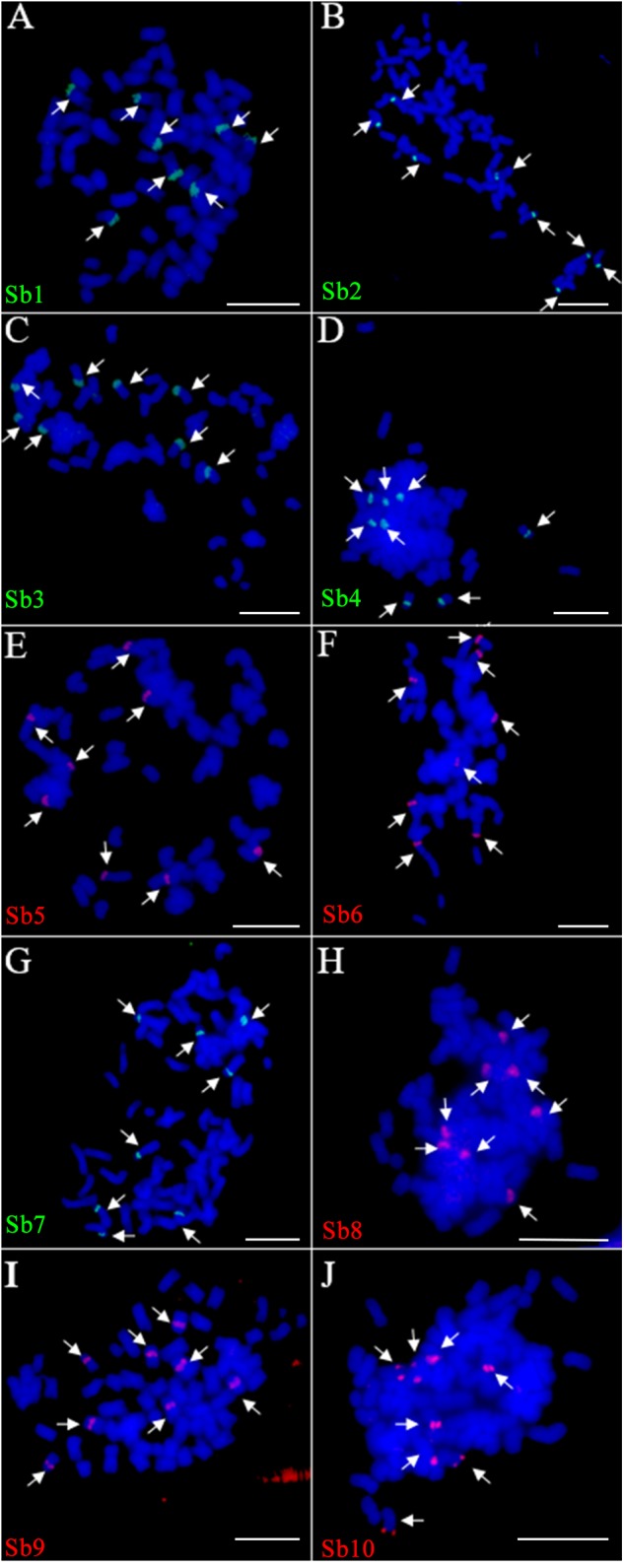
Fluorescence *in situ* hybridization (FISH) assay of the sorghum-derived oligo probes in *Saccharum spontaneum* SES208. **(A–J)** The 10 oligo probes derived from sorghum chromosome 1–10 (Table 1) were hybridized to the mitotic metaphase chromosomes of *S. spontaneum* SES208, respectively. Bright and clear signals were detected from each of the probes in SES208. The arrows in each cell indicate the FISH signals in the eight homologous chromosomes in octoploid *S. spontaneum* SES208. Scale bars, 10 μm.

Due to the lack of a well-assembled sugarcane genome, we designed the oligo based on the sorghum assembly^3^. Ten oligo probes (Sb1–Sb10), one for each of the 10 sorghum chromosomes, were synthesized (Table 1). In total, these ten oligo probes consist of 57,982 59-nt oligos (average at 5,798 oligos per probe) and span 0.6–9.8 Mb of the sorghum genome, with the density ranging from 0.74–2.49 oligos per kilobase (Table 1). As expected, the FISH assay showed that each of these 10 oligo probes can produce consistently clear and unique signals in sorghum (Supplementary Figure S1). These probes were then examined in FISH in sugarcane *S. spontaneum*. Excitingly, we also observed clear single signals in the sugarcane chromosome for each probe. As shown in Figure 1, for each probe, we observed signals from eight chromosomes, i.e., the eight homologous chromosomes of the auto-octoploid sugarcane, *S. spontaneum* SES208 (2*n* = 8*x* = 64) (Figure 1). Thus, these oligo probes that can identify individual chromosomes in *S. spontaneum* SES208 chromosomes were used for further study.

**Table 1:** Characterizations of oligo probes designed based on the sorghum genome.

*Sorghum bicolor* Chromosome	FISH probes	Start position (bp)	Stop position (bp)	Region length (bp)	Number of oligos	Density (oligos/kb)
1	Sb1	69,018,879	76,895,592	7,876,714	6,272	0.80
2	Sb2	66,300,038	73,968,893	7,668,856	6,272	0.82
						
3	Sb3	160,932	9,953,015	9,792,084	8,000	0.82
						
4	Sb4	48,935,966	58,486,525	9,550,560	7,672	0.80
5	Sb5	61,545,789	69,794,127	8,248,339	6,272	0.76
						
6	Sb6	49,008,288	56,997,039	7,988,752	6,272	0.79
7	Sb7	52,607,631	58,997,869	6,390,239	4,704	0.74
8	Sb8	54,503,580	60,699,807	6,196,228	4,704	0.76
						
9	Sb9	10,639	7,995,315	7,984,677	6,272	0.79
						
10	Sb10	1,905,762	2,525,998	620,237	1,542	2.49

### Chromosome Rearrangement Between *S. spontaneum* and Sorghum

**FIGURE 2:**
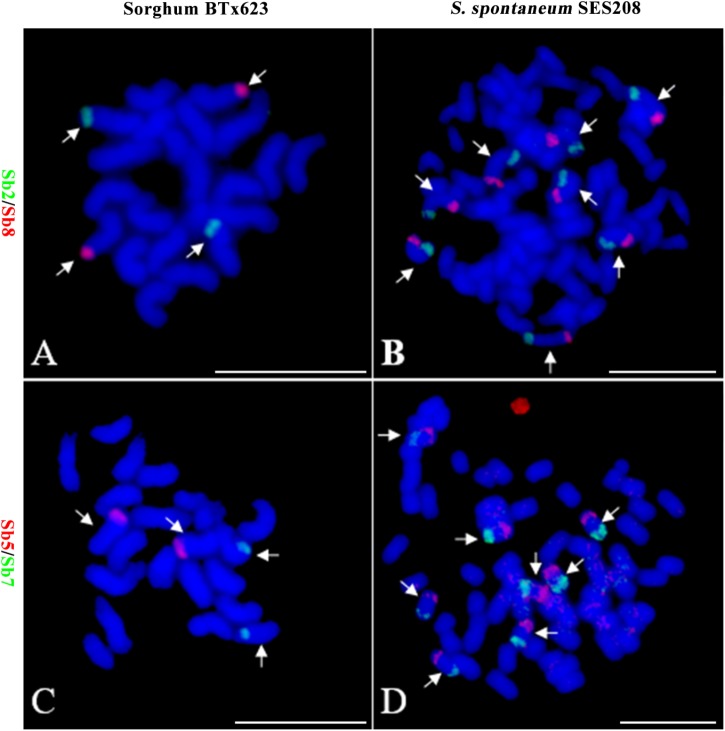
Validation of chromosomal rearrangement between sorghum and *S. spontaneum* by oligo-FISH. **(A,B)** A FISH assay of oligo probes Sb2 (green) and Sb8 (red) in sorghum BTx623 and *S. spontaneum* SES208. The FISH result confirmed that probes Sb2 and Sb8 are located on different chromosomes in sorghum **(A)** but are located at the opposite ends of the same chromosome in *S. spontaneum* SES208 **(B)**. **(C,D)** A FISH assay of oligo probes Sb5 (red) and Sb7 (green) in sorghum BTx623 and *S. spontaneum* SES208. The FISH result revealed that probes Sb5 and Sb7 are located on different chromosomes in sorghum **(C)** but are located at the opposite ends of the same chromosome in *S. spontaneum* SES208 **(D)**. The arrows indicate the chromosomes bearing FISH signals. Scale bars, 10 μm.

Because of the reduction of basic chromosomal numbers from 10 in sorghum to 8 in *S. spontaneum*, we hypothesized that chromosome fusion occurred in sugarcane after diverging from a common ancestor with sorghum. Thus, we anticipated that there were two or more sorghum-derived oligo probes concurring in one sugarcane chromosome. Through examining using a dual-color FISH, we found that the probes Sb2 and Sb8 (Figure 2A), which were derived from sorghum chromosomes 2 and 8, were located at opposite ends of the same chromosome in *S. spontaneum* SES208 (Figure 2B). In addition, probes Sb5 and Sb7, which were derived from sorghum chromosomes 5 and 7 (Figure 2C), were located at the opposite ends of the same chromosome in *S. spontaneum* SES208 (Figure 2D). These results indicate that chromosomal fusions between chromosomes 2 and 8, and between chromosomes 5 and 7, occurred and might have given birth to the two sugarcane chromosomes.

**FIGURE 3:**
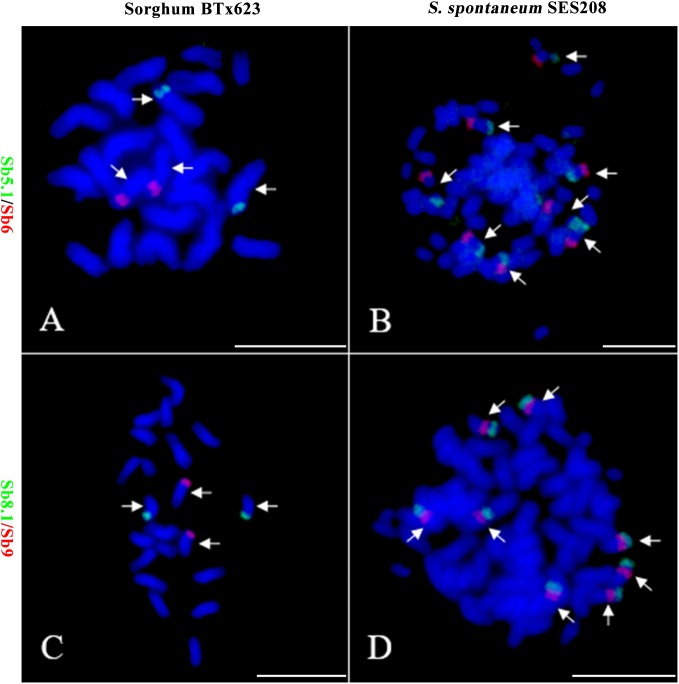
Further validation of chromosomal rearrangements between sorghum and *S. spontaneum* by oligo-FISH. **(A,B)** A FISH assay of oligo probes Sb5.1 (green) and Sb6 (red) in sorghum BTx623 **(A)** and *S. spontaneum* SES208 **(B)**. The FISH result confirmed that the probes Sb5.1 and Sb6 are located on different chromosomes in sorghum **(A)** but in the opposite ends of the same chromosome in *S. spontaneum* SES208 **(B)**. **(C,D)** A FISH assay of oligo probes Sb8.1 (green) and Sb9 (red) in sorghum BTx623 **(C)** and *S. spontaneum* SES208 **(D)**. The FISH result confirmed that probes Sb8.1 and Sb9 are located on different chromosomes in sorghum **(C)** but on the same chromosome in *S. spontaneum* SES208 **(D)**. Arrows indicate the chromosomes bearing FISH signals. Scale bars, 10 μm.

**FIGURE 4:**
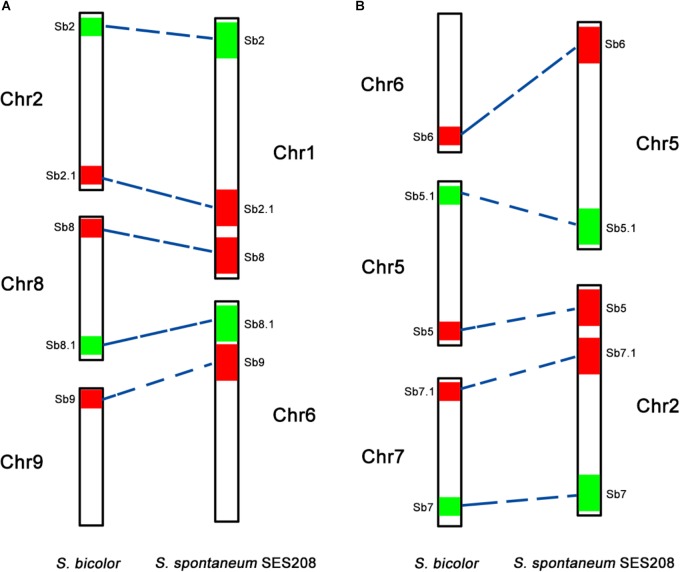
A schematic illustration of chromosomal rearrangements between sorghum and *S. spontaneum*. **(A,B)** Represent two chromosome rearrangement events involving sorghum chromosomes 2, 8, and 9, and sorghum chromosomes 6, 5, and 7, respectively. The chromosomes are depicted according to the oligo FISH results as shown in Figures 2, 3. The color bars represent the signals produced by the oligo probe. The relative length of each chromosome were drawn based on the data of the sorghum genome and Table 2.

To further assess the genomic regions that involve chromosome fusion, we developed four other probes Sb2.1, Sb8.1, Sb5.1, and Sb7.1, which were located at the opposite arms of probes Sb2, Sb8, Sb5, and Sb7 in sorghum, respectively (Supplementary Table S1 and Supplementary Figure S2). Then, we conducted dual-probes FISH using these four (i.e., Sb2.1, Sb8.1, Sb5.1, and Sb7.1) probes with the other ten probes (i.e., Sb1–10) one by one. The results showed that probe Sb5.1 and Sb6, which were derived from different sorghum chromosomes (Figures 3A, 4B), were located on the same chromosome in *S. spontaneum* SES208 (Figures 3B, 4B). In addition, probes Sb8.1 and Sb9, which were located on chromosomes 8 and 9 in sorghum (Figures 3C, 4A), were found to be located on the same chromosome in *S. spontaneum* SES208 (Figures 3D, 4A). For probes Sb2.1 and Sb7.1, the FISH results showed that they located on the same chromosome with probes Sb2 and Sb7 in SES208, respectively (Supplementary Figure S2). Therefore, we speculated that sorghum chromosome 8 was broken by an unknown rearrangement event, and that then Sb8 and Sb8.1-containing segments merged with chromosomes 2 and 9, respectively, giving birth to chromosomes 1 and 6 of *S. spontaneum*, respectively (Figure 4). Similarly, sorghum chromosome 5 was broken, and Sb5.1- and Sb5-involving segments broke away from chromosome 5 and were then combined with chromosomes 6 and 7 to generate the chromosomes 5 and 2 of *S. spontaneum*, respectively (Figure 4).

### Standard Karyotype Based on Chromosome Identification for *S. spontaneum* SES208

Based on the above results, we obtained a set of chromosome-anchored oligo probes that can be used to identify each of the eight chromosomes in *S. spontaneum* SES208 (Table 2). We then performed a FISH assay using the chromosome-anchored oligo probes to develop a standard karyotype for *S. spontaneum* SES208. A centromere-specific repeat DNA Ss51 (Zhang et al., 2017) was also used simultaneously in FISH to identify the centromere (Supplementary Figure S3). The karyotype data of *S. spontaneum* SES208 is listed in Table 2.

**Table 2:** Karyotyping and nomenclature of the mitotic chromosomes in *S. spontaneum* SES208.

*Saccharum spontaneum* chromosome	FISH probes	Long arm (μm)	Short arm (μm)	rDNA locations		Arm ratio^a^	Total length (μm)	Relative length^b^ (%)	*n*
				45S	5S				
1	Sb2, Sb8	2.36 ± 0.36	1.95 ± 0.26			1.21 ± 0.15	4.30 ± 0.57	14.50 ± 0.02	80
									
2	Sb5, Sb7	2.45 ± 0.51	1.62 ± 0.25			1.53 ± 0.32	4.06 ± 0.65	13.67 ± 0.02	80
3	Sb1	2.14 ± 0.32	1.88 ± 0.32			1.14 ± 0.12	4.02 ± 0.61	13.51 ± 0.02	80
									
4	Sb3	2.11 ± 0.31	1.77 ± 0.22			1.20 ± 0.14	3.88 ± 0.48	13.06 ± 0.02	80
									
5	Sb6, Sb5.1	2.19 ± 0.35	1.58 ± 0.33	Long arm		1.45 ± 0.38	3.77 ± 0.52	12.69 ± 0.02	80
									
6	Sb9, Sb8.1	2.23 ± 0.35	1.43 ± 0.23		Long arm	1.60 ± 0.28	3.65 ± 0.51	12.28 ± 0.02	80
									
7	Sb4	1.90 ± 0.29	1.61 ± 0.30			1.20 ± 0.20	3.52 ± 0.54	11.83 ± 0.02	80
									
8	Sb10	1.37 ± 0.20	1.15 ± 0.19			1.20 ± 0.17	2.52 ± 0.35	8.48 ± 0.01	80

*^a^Arm ratio, length of the long arm/length of the short arm. ^b^Relative length, chromosome length/genome length.*

**FIGURE 5:**
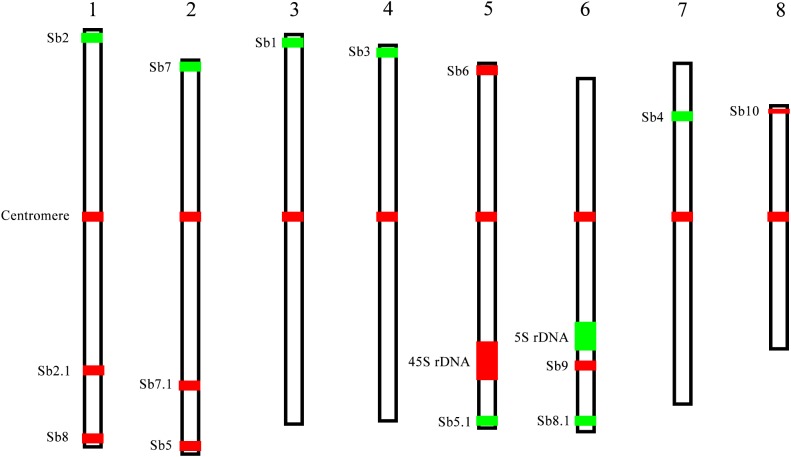
A representative chromosome schematic of *S. spontaneum* SES208. The color bars represent the signals produced by the probes used in this study. The relative length of each chromosome and signal positions were drawn based on the data in Table 2.

The karyotype data allow us to name the chromosomes in descending order of their lengths and follow the principle of chromosome nomenclature (Li and Chen, 1985; Slovak et al., 2013) (Table 2). The longest chromosome 1 is 4.30 μm long with a relative length of 14.50%; chromosome 8 is the shortest with a size of 2.52 μm and has a relative length of 8.48%. Chromosome 1 is only 1.7 times as long as the shortest chromosome 8. Therefore, none of the chromosome was exceptionally short or long, which is consistent with previous data (Ha et al., 1999). Based on the identification of centromeres using the centromere-specific probe, we could precisely calculate the chromosome arm. As a result, chromosome 6 has the largest arm ratio at 1.60, and chromosome 3 has the smallest arm ratio at 1.14. Thus, all of the chromosomes are metacentric (1.01 < arm ratio < 1.70) (Levan et al., 1964), and meaning that their arms were relatively equal in length (Table 2). To present an overview of the cytogenetic feature, an integrated schematic was drawn with the position of the 14 oligo probes, 45S rDNA, 5SrDNA and centromeres (Figure 5) based on its relative position (Table 2).

### Distributions of 45S and 5S rDNAs in *S. spontaneum* SES208

**FIGURE 6:**
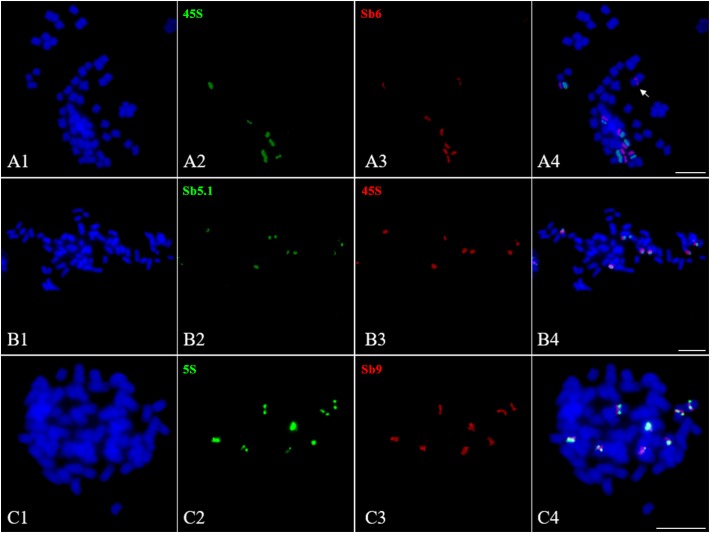
Fluorescence *in situ* hybridization mapping of 45S and 5S rDNAs in *S. spontaneum* SES208. **(A1–A4)**, **(B1–B4)**, and **(C1–C4)** Dual-probes FISH assay using probe pairs of 45S rDNA (green) and Sb6 (red), 45S rDNA (red) and Sb5.1 (green), and 5S rDNA (green) and Sb9 (red) in *S. spontaneum* SES208, respectively. **(A1–A4)** Show that the FISH signals of 45S rDNA and Sb6 are located at the opposite arms in chromosome 5 in *S. spontaneum* SES208. **(B1–B4)** Show that the FISH signals of 45S rDNA and Sb5.1 are located on the same arm in chromosome 5 in *S. spontaneum* SES208. **(C1–C4)** display that the FISH signals of 5S rDNA and Sb9 are located closely each other in the same arm in the same chromosome in *S. spontaneum* SES208. The arrow in **(A4)** indicates that one of the chromosome 5 homologous has no 45S rDNA signal. Scale bars, 10 μm.

The 45S and 5S rDNAs have highly conserved repetitive sequences in plants and are thus considered as an excellent markers for karyotype analysis (Deng et al., 2012). However, in *Saccharum*, due to a lack of an effective chromosome identification system, their chromosomal distributions remain unknown. To address this issue, we conducted a FISH assay in *S. spontaneum* SES208 using 45S rDNA and 5S rDNA combined with the probes of chromosome-specific oligos. Intriguingly, the FISH results show that there were seven chromosomes containing an observable 45S rDNA signal in *S. spontaneum* SES208 (Figure 6A2). A dual-probe FISH using 45S rDNA and chromosome-specific probes revealed that the 45S rDNA was located on the same chromosome (opposite arms) as probe Sb6, i.e., chromosome 5 in *S. spontaneum* SES208 (Figures 6A1–A4 and Table 2). Moreover, two of these seven signals showed apparently lower intensities, indicating that copy number variation occurred between these seven 45S rDNA loci (Figure 6A2).

Our results demonstrate that the 45S rDNA locus was missing from one of the eight homologous chromosomes of chromosome 5 in the autopolyploid sugarcane (Figures 6A1–A4). Because the probe Sb6 is located at the opposite arm from the 45S rDNA, we hypothesized that a chromosomal segment deficiency or translocation might have occurred in chromosome 5, causing the loss of the 45S rDNA. To test this hypothesis, we conducted FISH using another oligo probe Sb5.1, which was located at the opposite arm from Sb6 (Figure 3B), thus on the same arm as 45S rDNA. The FISH results confirmed that Sb5.1 was located closely to the 45S rDNA in the same chromosome in *S. spontaneum* SES208 (Figures 6B1–B4). However, one of the 45S rDNA is still absent from one of the Sb5.1-bearing chromosomes. Thus, our results suggest that the loss of this 45S rDNA from one of the homologous chromosomes of chromosome 5 was caused by an unknown mechanism other than a large chromosomal segment (involving the proximate segment of Sb5.1) deletion or translocation.

Eight 5S rDNA FISH signals were detected in *S. spontaneum* SES208, and each chromosome has one signal (Figures 6C2,C4). A dual-probe FISH revealed that the 5S rDNA was located on the same chromosome as probe Sb9, i.e., chromosome 6 (Figures 6C1–C4). Similar to in 45S rDNA, significant differences in signal intensities were also detected for these 5S rDNAs (Figure 6C2), indicating a high level of copy number variations between these 5S rDNA loci.

## Discussion

Great efforts have been made in studying the genetics, genomics and breeding of sugarcane because of the importance of sugarcane to sugar production. However, the complexity of the genome, including the large chromosomal number and high level of heterozygosity, has hindered severely progress in genetic/genomics research of sugarcane. Cytogenetics, including classical cytogenetics and molecular cytogenetics, have played an essential role in the genomic studies of sugarcane (Sreenivasan et al., 1987; Ming et al., 2010). However, reliable cytogenetic markers for identifying individual chromosomes in sugarcane are still unavailable, which hinders deeply deciphering the structure and evolution of the sugarcane genome. Recently, we have conducted a trial to screen the BAC of sugarcane and to use BAC clones as the marker for chromosome identification (Dong et al., 2018). However, the high level of repetitive sequences (77.4%) (Zhang et al., 2017) caused high background noise in BAC-FISH (Dong et al., 2018), which is inevitable in FISH analyses of species having a high level of repetitive DNAs (Jiang and Gill, 2006). Even worse, the sequence variation between homologous chromosomes also caused varied signal intensities, some of which were too weak to be detected (Dong et al., 2018). In this study, we developed sorghum oligo probes spanning several megabases and much larger than the inserted genomic fragments in BAC (usually ∼100 kb). Thus, these probes can produce bright signals in FISH. Moreover, benefiting from the removal of repetitive DNAs, these probes can produce clear signals with nearly no background noise in FISH in both sorghum and sugarcane. As shown in FISH (Figure 1 and Supplementary Figure S1), each chromosome and its homologous chromosomes can be unambiguously identified based on the detection of FISH signals derived from our oligo probes.

The development of chromosome-specific oligo probes provides us a powerful tool in the study of the structure and evolution of a genome. In fact, being able to identify individual chromosomes has opened a new door for cytological studies of species having large and complex genomes, such as in the case of the *Saccharum* genus. Based on the precise identification of individual chromosomes, we obtained, for the first time, standard karyotype data for the autopolyploid *S. spontaneum*, which is the most primitive species in the genus *Saccharum* (Stevenson, 1965; Ming et al., 2010). Consistently, the nearly equal signal intensities derived from the eight homologous chromosomes for each probe (Figure 1) agree with the notion of an autopolyploid nature for this species. Interestingly, we revealed that both 45S and 5S rDNAs displayed copy number variations among their homologies, 45S was absent even in one chromosome. Although the mechanism causing the copy number variation and the missing of one rDNA loci is unknown, it at least indicates that the rDNAs underwent a more rapid evolution than the unique DNAs between homologous chromosomes in sugarcane.

The clear signals in *S. spontaneum* produced by the sorghum oligo probes demonstrate a high level of sequence homology, which is consistent with the previous result based on DNA sequence analyses (average 95.2% sequence identity) (Wang et al., 2010). However, a potential problem is whether our oligo probes derived from genus of *Sorghum* are applicable for other species of *Saccharum*. Previous studies in *Cucumis* suggest that the probes developed from cucumber genome sequences can be used in a related species that diverged as long as 12 MYA (Han et al., 2015). Based on the sequence analyses, sugarcane and sorghum are suggested to have diverged from a common ancestor ∼7 MYA (Jannoo et al., 2007; Wang et al., 2010; Dong et al., 2018), and the divergence among different species in *Saccharum* has been proposed to be less than 2 MYA (Jannoo et al., 2007; Zhang et al., 2018). Thus, it suggests that this set of oligo probes can be used in all of the species in *Saccharum.*

Chromosomal rearrangement, including chromosome fusion, chromosomal translocation, and chromosomal deficiency has played a key role in the genomic evolution of both plants and animals (Lysak et al., 2006; Schubert, 2007; Wijchers and de Laat, 2011; Geiser et al., 2016). For example, in *Arabidopsis thaliana* and the related *Brassicaceae* species, the reciprocal translocation and elimination of minichromosomes have reduced the basic chromosome number from 8 to 5 (Lysak et al., 2006). Although having a close relationship, *Sorghum bicolor* and *S. spontaneum* have different basic chromosome numbers of 10 and 8, respectively (Daniels and Roach, 1987; D’Hont et al., 1998; Ming et al., 2010). In this study, we reveal that chromosome rearrangement events involving the sorghum chromosomes 2, 8, and 9, and chromosomes 5, 6, and 7 (Figure 4), likely contributed to the basic chromosome number reduction from 10 to 8 between sorghum and *S. spontaneum.* Thus, our results provide the first cytological evidence for the basic chromosome number reduction from 10 to 8 in sorghum and *S. spontaneum*. Further studies with more probes or the “chromosome painting” probe will gain deeper insights on the chromosome rearrangements and uncover the karyotype evolution between or within these two species.

An important use for the chromosome-specific markers is for investigating genomic evolution by comparing the chromosomal collinearity between different species within a genus or relative genera. Based on a DNA sequence comparison, Kim et al. (2014) suggested that the *Saccharum* and *Miscanthus* shared an allopolyploid event before the divergence of these two genera approximately 3.8–4.6 MYA. Most recently, Zhang et al. (2018) detected 71 interchromosomal rearrangements between sorghum and *S. officinarum* or *S. robustum* based on the large scale of SNPs analyses. Interestingly, 24 (33.8%) interchromosomal rearrangements were shared by *S. officinarum* and *S. robustum* (Zhang et al., 2018), indicating that the common ancestor of *S. officinarum* and *S. robustum* was a diploid and thus was unlikely to have shared an allopolyploid event before the divergence of *Saccharum* and *Miscanthus* (Zhang et al., 2018). This contradiction was likely caused by the complexity of polyploid Saccharum genome and the limited plant sample used. However, an issue for the sequence comparison is the limitation of DNA sequence data available for species with a large and complex genome, such as in *Saccharum*. Fortunately, the oligos developed in this study could be used as cross-genus probes to detect chromosomal collinearity for *Sorghum* and *Saccharum* or for relative genera without the requirement of DNA sequences. Thus, the cytological analysis by oligo-FISH provides us an alternative tool for the study of chromosome evolution. Further studies using these probes at a wider scale of the species, from the genera of *Sorghum* and *Saccharum* and their relative genera will provide us deep insights into the evolution of their genomes.

## Conclusion

Using a massive oligo synthesis strategy, we developed a complete set of chromosome-specific oligo probes for the large and complex genome sugarcane species, *S. spontaneum*. We demonstrated that these probes can produce clear signals in corresponding chromosomes and that each of the chromosomes in *S. spontaneum* can be unambiguously identified. Moreover, these probes could be used as cross-species markers for cytological analyses between the species in both *Sorghum* and *Saccharum*. By comparing FISH analyses, we found that chromosome rearrangement events might have contributed to the basic chromosome number reduction from 10 in sorghum to 8 in sugarcane. Therefore, the creation of this set of chromosome-specific probes remarkably improve our ability to conduct cytological research on sugarcane, and applying such probes to future studies will bring us deep insights into the genomic structure and evolution of sugarcane.

## Author Contributions

KW, ZM, and YH conceived the study and drafted manuscript. ZM, TY, YW, YH, and WH conducted the cytogenetic experiments. ZZ and KW designed the chromosome-specific oligo probes. KW, ZM, TY, YW, WH, QY, JW, ZL, and ZZ participated in the data analysis and manuscript preparation. All authors read and approved the final manuscript.

## Conflict of Interest Statement

The authors declare that the research was conducted in the absence of any commercial or financial relationships that could be construed as a potential conflict of interest.

## Footnotes

1 https://github.com/forrestzhang/Chorus

2 http://www.repeatmasker.org/

3 https://www.ncbi.nlm.nih.gov/assembly/GCF_000003195.3
