# Supplementary figures and images for: Comprehensively Characterizing the Cytological Features of Saccharum spontaneum by the Development of a Complete Set of Chromosome-Specific Oligo Probes
Source: Front Plant Sci. 2018 Nov 6;9:1624. doi: 10.3389/fpls.2018.01624 (PMC6232525; doi:10.3389/fpls.2018.01624)

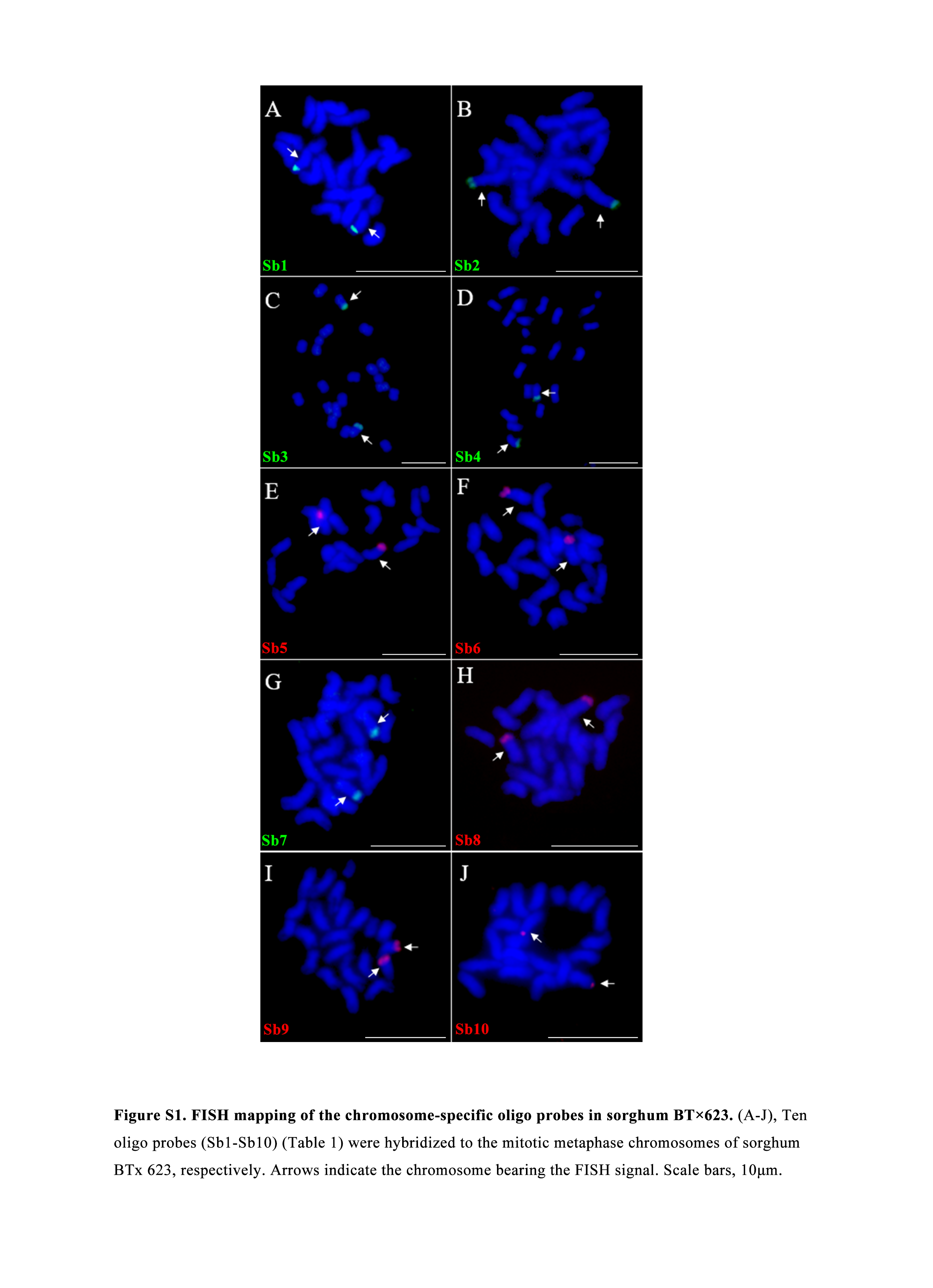

Supplement: Supplementary file 1 [file Image_1.TIF]

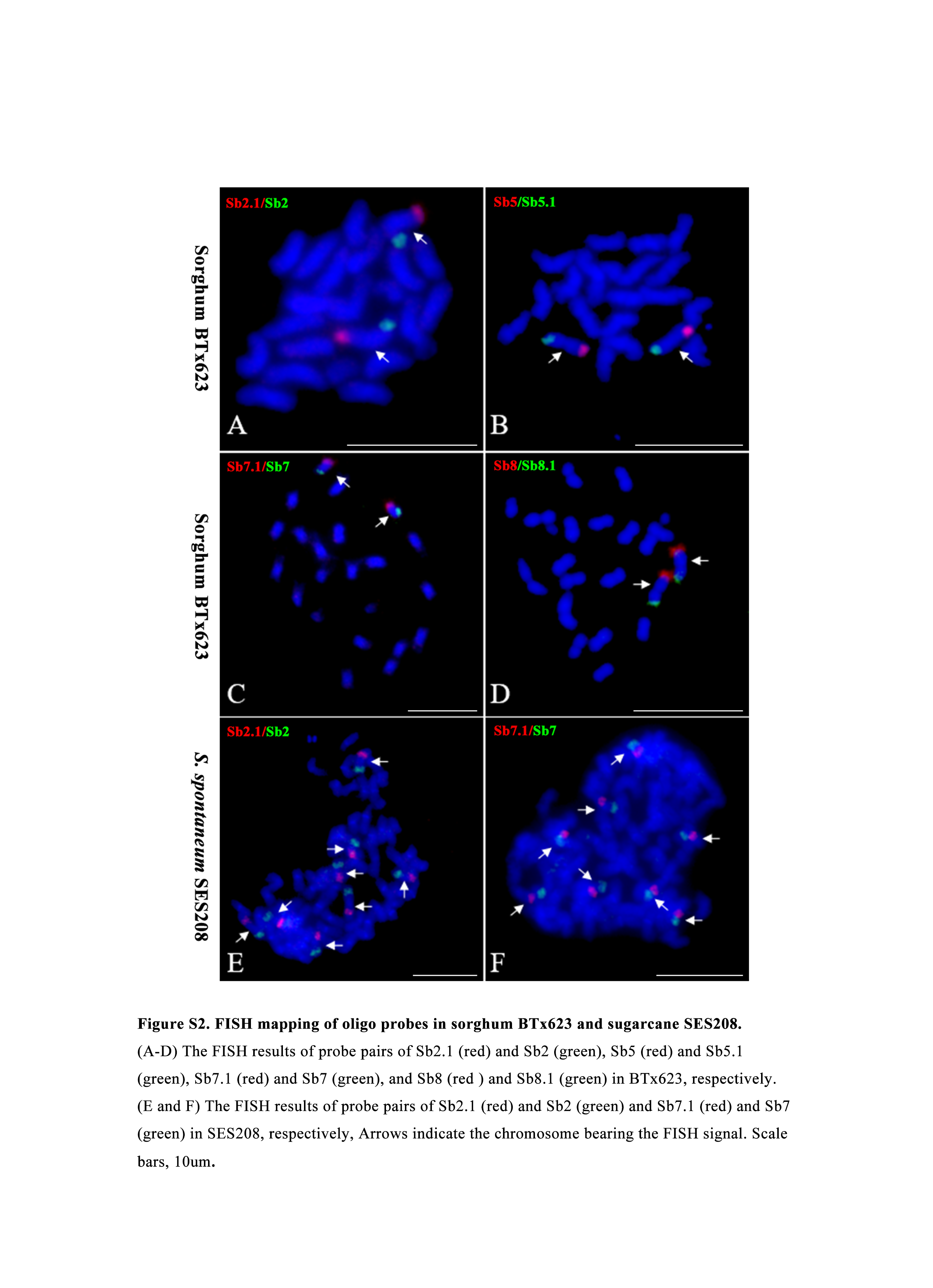

Supplement: Supplementary file 2 [file Image_2.TIF]

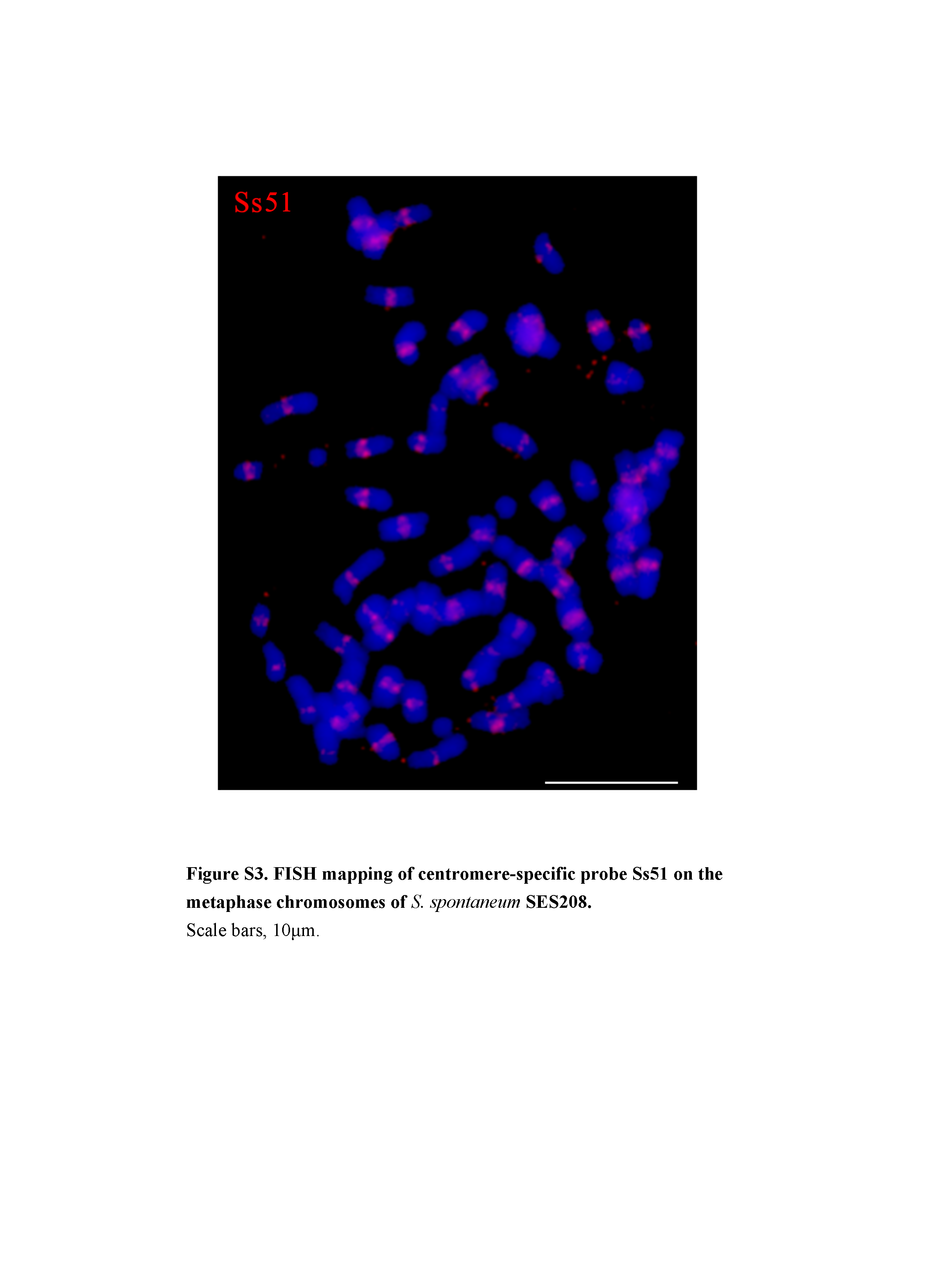

Supplement: Supplementary file 3 [file Image_3.TIF]
