# Supplementary material for: Comprehensively Characterizing the Cytological Features of Saccharum spontaneum by the Development of a Complete Set of Chromosome-Specific Oligo Probes
Source: Front Plant Sci. 2018 Nov 6;9:1624. doi: 10.3389/fpls.2018.01624 (PMC6232525; doi:10.3389/fpls.2018.01624)
Supplement: Supplementary file 4 [file Table_1.DOCX]

**Table S1. Characterizations of oligo probes Sb2.1, Sb 5.1, Sb 7.1 and Sb 8.1.**

| ***S.bicolor***  **Chromosome** | **FISH probes** | **Start position (bp)** | **Stop position (bp)** | **Region length (bp)** | **Number of oligos** | **Density** |
| --- | --- | --- | --- | --- | --- | --- |
| 2 | Sb2.1 | 63,334 | 5,969,141 | 5,905,808 | 4,704 | 0.80 |
| 5 | Sb5.1 | 2,000,417 | 7,987,228 | 5,986,812 | 4,704 | 0.79 |
| 7 | Sb7.1 | 2,000,155 | 9,798,478 | 7,798,324 | 6,272 | 0.80 |
| 8 | Sb8.1 | 2,000,071 | 11,574,191 | 9,574,121 | 6,272 | 0.66 |
